# QDPR homologues in *Danio rerio* regulate melanin synthesis, early gliogenesis, and glutamine homeostasis

**DOI:** 10.1371/journal.pone.0215162

**Published:** 2019-04-17

**Authors:** Maximilian Breuer, Luca Guglielmi, Matthias Zielonka, Verena Hemberger, Stefan Kölker, Jürgen G. Okun, Georg F. Hoffmann, Matthias Carl, Sven W. Sauer, Thomas Opladen

**Affiliations:** 1 University Children's Hospital, Division of Child Neurology and Metabolic Diseases, Heidelberg, Germany; 2 Heidelberg University, Medical Faculty Mannheim, Department of Cell and Molecular Biology, Mannheim, Germany; 3 University of Trento, Department of Cellular, Computational and Integrative Biology (CIBIO), Laboratory for Translational Neurogenetics, Trento, Italy; Wayne State University School of Medicine, UNITED STATES

## Abstract

Dihydropteridine reductase (QDPR) catalyzes the recycling of tetrahydrobiopterin (BH_4_), a cofactor in dopamine, serotonin, and phenylalanine metabolism. QDPR-deficient patients develop neurological symptoms including hypokinesia, truncal hypotonia, intellectual disability and seizures. The underlying pathomechanisms are poorly understood. We established a zebrafish model for QDPR deficiency and analyzed the expression as well as function of all zebrafish QDPR homologues during embryonic development. The homologues qdpra is essential for pigmentation and phenylalanine metabolism. Qdprb1 is expressed in the proliferative zones of the optic tectum and eye. Knockdown of qdprb1 leads to up-regulation of pro-proliferative genes and increased number of phospho-histone3 positive mitotic cells. Expression of neuronal and astroglial marker genes is concomitantly decreased. Qdprb1 hypomorphic embryos develop microcephaly and reduced eye size indicating a role for qdprb1 in the transition from cell proliferation to differentiation. Glutamine accumulation biochemically accompanies the developmental changes. Our findings provide novel insights into the neuropathogenesis of QDPR deficiency.

## Introduction

Dihydropteridine reductase (human: DHPR; mouse / zebrafish: Qdpr) is the key recycling enzyme of the cofactor tetrahydrobiopterin (BH_4_). The homodimer uses NADH to supply two hydrogen atoms to BH_2_ to recover BH_4_ [1]. BH_4_ is initially formed in a three-step pathway *de-novo* from GTP and is then salvaged in a recycling pathway via pterin-4a-carbinolamine dehydratase (PCBD) and DHPR [2, 3]. This pathway is highly conserved among species [4–6]. The zebrafish genome contains three DHPR homologs, Qdpra, Qdprb1 and Qdprb2, the function of which has remained largely unknown.

BH_4_ is cofactor in the enzymatic reaction of phenylalanine hydroxylase (PAH), tyrosine hydroxylase (TH) and tryptophan hydroxylase (TPH). PAH catalyzes the formation of tyrosine from phenylalanine and is therewith essential for phenylalanine degradation and synthesis of the neurotransmitter precursors L-dopa through TH, whereas TPH is responsible for serotonin biosynthesis. The BH_4_ pathway is also needed for the activity of nitric oxide synthases (NOS) [2] and has been linked various clinical entities including autism [7], pain regulation [8] and cardiovascular diseases [9].

Hyperphenylalaninemia (HPA) due to inborn defects of PAH is the biochemical hallmark of phenylketonuria (PKU). In the majority of BH_4_ deficiencies HPA also arises reflecting impaired PAH function due to shortage of BH_4_ and is treated by diet or with BH_4_ (sapropterin dihydrochloride) [10]. In contrast to PKU, BH_4_ deficiencies are complicated by depletion of dopamine and serotonin, which can be overcome by oral supplementation with the neurotransmitter precursors L-Dopa and 5-HT [11].

In comparison to other BH_4_ deficiencies, DHPR-deficient patients show a higher frequency of severe neurological symptoms including hypotonia, dystonia, microcephaly, epilepsy and brain atrophy [11, 12]. Due to deficient DHPR activity 7,8-dihydrobiopterin (BH_2_) accumulates that has been suggested to play a significant role in the neuropathogenesis of affected patients by inhibiting nitric oxide synthase and aromatic acid hydroxylases [13]. Oral BH_4_ supplementation as recommended in other BH_4_ deficiencies is likely to aggravate BH_2_ accumulation and, therefore, dietary restriction of phenylalanine intake to prevent HPA is the treatment of choice in DHPR-deficient patients. Qdpr-knockout mice have been generated to better understand the pathophysiology of DHPR-deficiency. Indeed, these mice show similar biochemical alterations including HPA and BH_2_ accumulation and, additionally, have a higher sensitivity to oxidative stress [5]. However, these animals do not develop a neurological phenotype. Unlike in humans, mice have a high cerebral dihydrofolate reductase (Dhfr) expression. This protein is essential for homeostasis of folates and can also recycle BH_2_ [14]. High cerebral Dhfr activity in Qdpr-deficient mice may consequently explain the apparent lack of a neurological phenotype [5].

Since *Qdpr* knockout mice do not develop a clinical phenotype similar to DHPR-deficient patients, we aimed to establish a novel animal model to study the pathophysiology of DHPR deficiency. For the study of genetic diseases including inborn, metabolic disorders, zebrafish have become increasingly valuable as many proteins are conserved in their function, developmental changes can be observed *ex utero*, and their genome and gene expression can easily be manipulated. In fact, an increasing number of inborn errors of metabolism are identified and characterized in vivo via gene manipulations in zebrafish [15, 16]. BH_4_ metabolism has previously been studied in zebrafish, yet only regarding its role in pigment synthesis [6]. The de novo synthesis pathway of BH_4_ generates pterin precursors for the yellow pigments in zebrafish eyes [6, 17]. The dark pigment melanin is generated from tyrosine via L-dopa and eumelanin. Since this pathway is initiated by Th, melanin formation depends on de novo synthesis and recycling of BH_4_. Accordingly, down regulation of Pcd catalyzing the first step of BH_4_ recycling lead to reduced melanin production in zebrafish [18, 19].

The aim of this study was to analyze the DHPR zebrafish homologues (Qdpra, Qdprb1, Qdprb2) and their role in zebrafish development. We show that Qdpra is involved in the regulation of phenylalanine degradation and pigment synthesis. Qdprb1 on the other hand is essential for early gliogenesis and regulation of the glutamine homeostasis shining new light onto the pathophysiology of DHPR deficiency.

## Material and methods

### Fish husbandry

Zebrafish were maintained at 28°C at a 14/10 h light/dark cycle and in accordance to [20]. Zebrafish were held in accordance with all international and national laws and obligations as registered at the Regierungspräsidium Karlsruhe (Az. 35–9185.81/G-85/16). Embryos were raised in E3 medium at 28.5°C until desired [21]. To inhibit pigment synthesis, embryos were kept in 0.003% 1-phenyl-2-thiourea (PTU) (Sigma) in E3 medium after gastrulation. AB wildtype fish and the following transgenic lines were used in this study: tg(HUC/D:GFP; Park [22] and tg(NBT/lyn:GFP).

The regional council (Regierungspräsidium Karlsruhe, Baden-Württemberg, Germany) prospectively approved this zebrafish research (Approval number 35–9185.81/G-85/16). Data from one patient with DHPR deficiency was collected within the iNTD study, approved by the local ethic board of the Medical Faculty in Heidelberg (Approval number S-471/2014) [23].

### MO injections and rescue experiments

Morpholinos were designed and provided by GeneTools LCC (Oregon, USA). 4.5 ng of qdpra splice blocking (5’-CTTAGGTGTCCTAACCTTTCGAGCT-3’) and 4.6 ng qdprb1 splice blocking MO (5’-TATTAGGCGAGTACCAACTTTTGGC—3’) were injected at the 1-cell stage, while for qdprb1 ATG MO (5’-TAGCTGCCATTCTGTCTTCACGAGC—3’) 1.5 ng were injected. Control MOs with a 5bp mismatch were used to validate no developmental delay. Furthermore, in accordance with Ekker and Larson [24], we confirmed a synergistic effect of the MOs at low concentrations shown in Figure D in S3 File. Rescues were performed with 80 pg/μl whole mRNA fish *qdprb1* (NM_001020698.1) and 500 pg/μl mouse transcript variant 001 (NM_024236.2). mRNA was *in vitro* transcribed in *pcs2+* vector using Sp6 mMessage Machine Kit (Ambion) and co-injected with the respective MOs.

### Whole mount *in situ* hybridization

WISH was performed as shown by [25]. Minor modifications included incubation at 70°C rather than 60°C. DIG-labelled *in situ* hybridization antisense-mRNA probes were *in vitro* transcribed from the linearized PcrII dual promotor (ThermoFisher) vector containing the respective gene or gene fragment. Sense probes were used as control. Individual probes were generated for *gch1*, *gfap*, *glula*, *otx2*, *pah*, *qdpra*, *qdprb1*, *qdprb2*, *slc1a2a*, *slc1a2b*, *slc1a3a*, *slc1a3b*, *wnt1*.

### Whole mount fluorescent immunohistochemistry

Whole mount fluorescent immunohistochemistry was performed as described previously [26]. Embryos were incubated overnight with primary antibody (pAb Rabbit Anti-phospho-Histone H3 (Ser10) (Millipore, Cat# 06–570) and, after a washing step, incubated overnight with secondary antibody (Cy2 AffiniPure Goat Anti-Rabbit IgG (H+L), Cat# 111-225-144) or DAPI. Secondary antibody was washed off and embryos were used for imaging. For fluorescence confocal microscopy, embryos were mounted in 1% low-melt agarose in glass-bottom dishes (MatTek or LabTek). Embryos were imaged using A TCS SP5 MP (Leica) inverse laser scanning microscope. Subsequent image analysis was performed with Fiji software.

### Real time–quantitative PCR

RNA was isolated from 25–30 embryos of each condition from the same experiment using Trizol according to manufacturer’s protocol. 500 ng isolated RNA was reverse transcribed via First Strand Maxima Synthesis kit (ThermoFisher). RT-qPCR was performed using SYBR Green Mix (Bioline) with previously tested primers on a BioRAD CFX Connect. Tests were run in duplicates of at least three independent experiments. Expression was normalized to Elongation factor 1 alpha expression. S1 Table shows the list of studied genes and used primer pairs.

### Melanin quantification via spectrophotometry

Melanin content was determined as shown by [27, 28]. Zebrafish were lysed in 1.0M NaOH with 10% DMSO. Lysate was incubated at 80°C for 1 hour and absorbance measured at 475nm. Wildtype were set as 100%.

### Biochemical measurements and exposures

Lysates from zebrafish embryos in water were used for analysis of amino acids. Embryos were lysed and homogenized on ice and in water by repeated passing through a 27G hypodermic needle (Terumo) and sonification using a “Branson 450 Sonifier” at 50% duty for 10 pulses with three repeats. Samples were stored at -20°C until measurement. Deproteinization was done by addition of 20% sulfosalicylic acid and precipitates were removed by centrifuging samples at 13,000rpm for 30 minutes. Amino acids were analyzed by Biochrom 30^+^ Cation exchange chromatography. For glutamine exposures, embryos were exposed to 1 or 20mM L-Glutamine in E3 medium. Medium was changed daily. Exposure from sphere stage until 72 hpf was considered an early developmental hyperglutaminemia and exposure from 48 hpf until 72 hpf was considered a late developmental hyperglutaminemia.

### Glutamine measurement in CSF of a patient with DHPR deficiency

Glutamine in CSF was measured during diagnostic work-up in a patient with DHPR deficiency as described previously [29]. At the time of lumbar punction the patient was not on medical treatment.

### Statistics

All experiments were repeated for a minimum of three biological replicates. Single comparisons were analyzed via student’s t-test and multiple comparisons using one-way ANOVA with post hoc Bonferroni and Holm evaluation. Values of p<0.05 were considered to be significant*.

## Results

The zebrafish genome contains three annotated QDPR homologues, Qdpra, Qdprb1 and Qdprb2. Therefore, we tested their function individually.

### *Qdprb2* analysis

*Qdprb2* is the homologue with the lowest homology to human DHPR with 62% amino acid conservation. *Qdprb2* expression was not detected in whole mount *in situ* hybridization (WISH). RT-qPCR analysis showed very transient maternal *qdprb2* expression (Figure A in S1 File). Consistently, functional knockdown with a *qdprb2* specific Morpholino (MO) resulted in no morphological phenotype. We concluded that *qdprb2* function has diverged during evolution and did not retain a functional role similar to its mammalian counterpart.

### Qdpra regulates BH_4_-related phenylalanine and melanin metabolism

**Fig 1:**
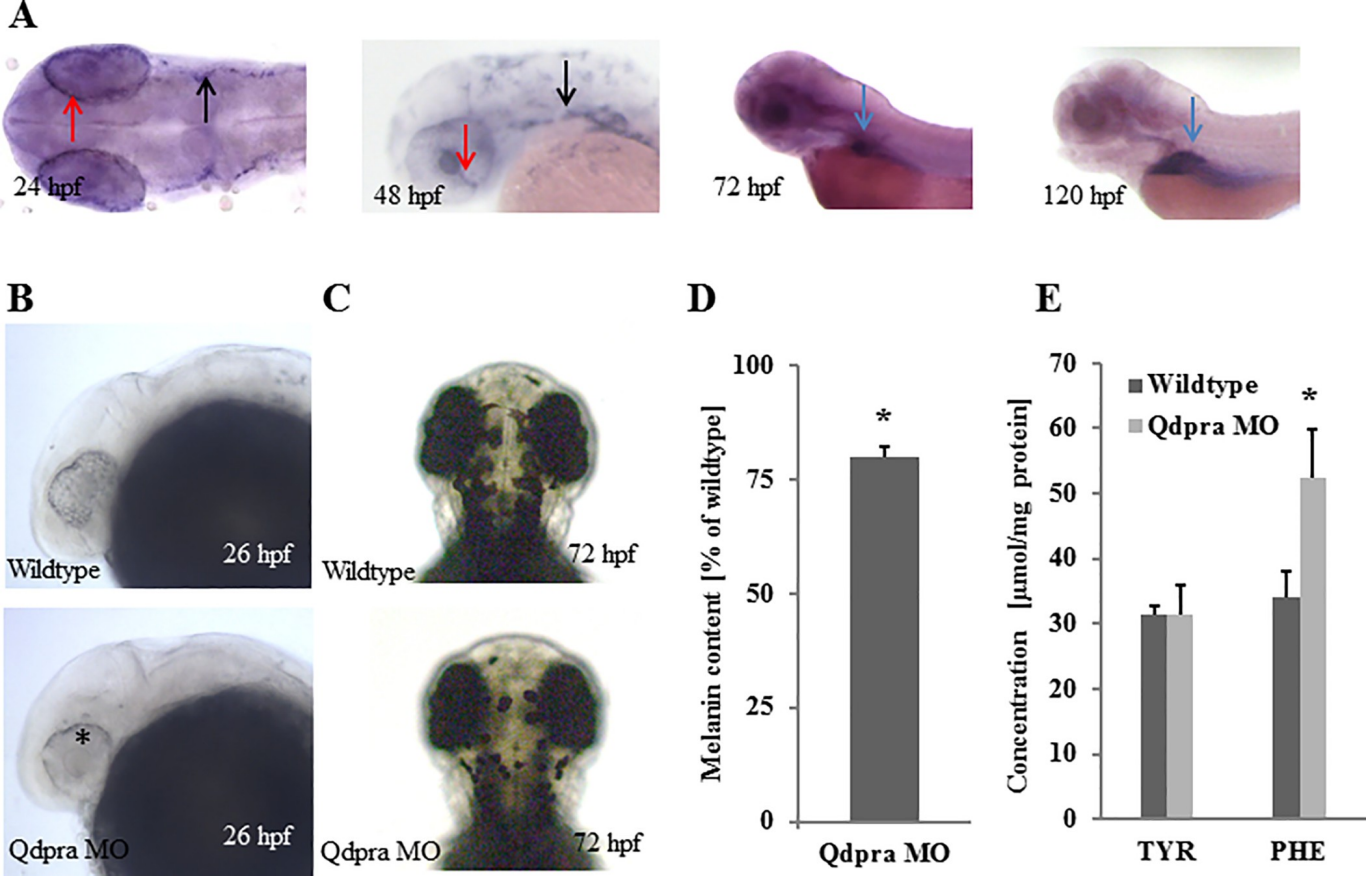
Characterization of Qdpra. (A) WISH of *qdpra* at 24 hpf (dorsal view, anterior to the left) shows staining in retinal pigment epithelium (red arrow) and neural crest cells/melanophore precursor (black arrow). At 48 hpf *qdpra* transcripts are found in the retinal pigment epithelium, choroid fissure (red arrow) and neural crest cells (black arrow). At 72 hpf and more pronounced at 120 hpf (lateral views, anterior to the left), staining is present in the liver (blue arrow). (B) Lateral views with anterior to the left and (C) dorsal views with anterior to the top of embryos at stages indicated. Knockdown of *qdpra* results in reduced pigments in the eye (asterisk) at 26 hpf (B) and overall diminished pigmentation at 72 hpf (C). (D) At 72 hpf melanin content is reduced by 20% (of wildtype) in Qdpra hypomorphic zebrafish. (E) Amino acid analysis shows hyperphenylalaninemia and normal tyrosine upon *qdpra* knockdown.

The homologue *qdpra* has 72% homology to the human DHPR protein. As shown by whole mount WISH, it is first visible at 18 hours post fertilization (hpf) in the eye and neural crest derived pigment cells, which is more apparent at 24 hpf when *qdpra* is also expressed in the retinal pigment epithelium of the eye (Fig 1A). At 48 hpf the expression is detected in developing melanophores along the axis of the body, as well as in the retinal pigment epithelium and choroid fissure of the eye. At 72 hpf, *qdpra* expression in the eye is decreasing, while expression in the liver arises. At 120 hpf, only the liver retains *qdpra* expression (Fig 1A). RT-qPCR levels show a rise in *qdpra* expression at 20 hpf coinciding with the early production of melanin in melanophores [30], and, later on, expression levels increase at 72 hpf, co-inciding with the expression of *Pah* in the liver (Figure B in S1 File, Figure A in S2 File).

*Qdpra* knockdown using a splice blocking MO did not result in visible morphological changes of the embryo. RT-PCR confirmed the predicted excision of exon 3 (Figure B in S File). During the rapid increase of melanophore pigmentation at around 26 hpf treated fish appear pale (Fig 1B), which becomes fully apparent at 72 hpf, when pigmentation and melanin content is strongly reduced compared to controls (Fig 1C and 1D). Injection of *qdpr* mRNA from mouse restored melanin pigmentation confirming the specificity of the effect (Figure C in S2 File).

Next, we analyzed the biochemical phenotype of *qdpra* hypomorphs at 72 hpf. DHPR-deficient patients typically display phenylalanine accumulation [11]. Downregulation of *qdpra* similarly resulted in an increase of phenylalanine levels in zebrafish (Fig 1E). Similar to *qdpr* knockout mice [5], we detected a depletion of taurine in *qdpra* morphants (wt: 24,39 +/- 0,35 μmol/mg; Qdpra MO: 20.67 +/- 0.16 μmol/mg; p<0.01). All other amino acids analyzed remained unchanged.

In summary, our findings indicate that Qdpra is necessary for melanin synthesis in melanophores. Biochemical analyses as well as region-specific and temporal expression profiles of *qdpra* suggest a role in hepatic phenylalanine metabolism.

### Qdprb1 influences brain development

**Fig 2:**
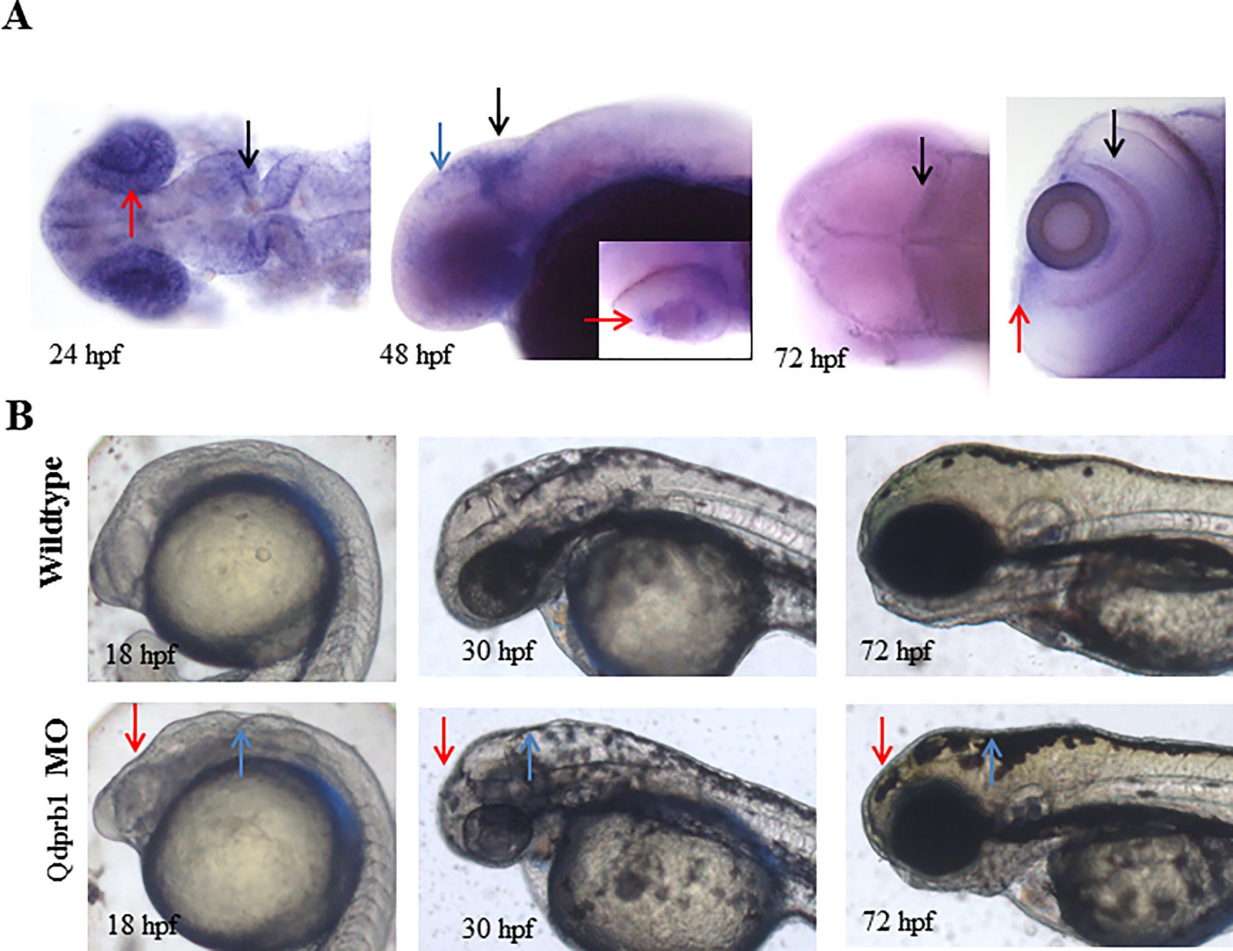
Characterization of Qdprb1. (A) WISH of *qdprb1* at 24 hpf (dorsal view, anterior to the left) shows staining in eye (red arrow) and mid-hindbrain boundary (black arrow), at 48 hpf (lateral view, anterior to the left) in the optic tectum (blue arrow) and the mid-hindbrain boundary (black arrow) and CMZ (inset; red arrow, dorsal view, anterior to the left), and at 72 hpf (dorsal view, anterior to the left) in proliferative regions of the optic tectum (black arrow, left picture), CMZ (red arrow, right picture, dorsal view of the eye), as well as inner retinal layer (black arrow). (B) Lateral views, anterior to the left. Comparison of wildtype embryos at 18 hpf, 24 hpf and 72 hpf and qdprb1 hypomorphic embryos exhibit abnormal midbrain (red arrow) and anterior hindbrain (blue arrow) morphology and microcephaly.

We next characterized the homologue *qdprb1*, which shows also 72% homology to the human homologue, yet only 73% homology to Qdpra. After weak expression during gastrulation, *qdprb1* becomes mainly confined to the optic tectum (OT), eye and mid-hindbrain boundary (MHB) at 24 hpf (Fig 2A). At 48 hpf, *qdprb1* expression in OT and MHB is retained, while the eye localizes along the developing ciliary marginal zone (CMZ), which is the main area for proliferating cells in the retina. This CMZ expression is retained at 72 hpf, by which time *qdprb1* is also more confined to an area of proliferative cells in the OT [31] (Fig 2A). Additionally, *qdprb1* expression at 72 hpf is observed in the inner retinal layer. mRNA expression levels show a continuous expression throughout development (Figure C in S1 File).

Knockdown of *qdprb1* using splice blocking MO resulted in morphological abnormalities of the developing brain, first seen at around 18 hpf and affecting mid- and hindbrain. Morphants showed decreased brain size at 30 hpf and microcephaly at 72 hpf with aberrant mid- and hindbrain development (Fig 2B). Trunk and tail structures of *qdprb1* hypomorphic embryos remained unaffected.

Co-injection of *p53* MO [32] together with *qdprb1* MO did not rescue the observed phenotypes highlighting that they did not result from p53 mediated apoptosis due to *qdprb1* MO toxicity (Figure A in S3 File). *Qdprb1* MO specificity was further vigorously ascertained. RT-PCR analyses showed the predicted inclusion of intron 3 and RT-qPCR analyses the strongly reduced expression of correctly spliced mRNA (Figure B, C in S3 File). Injection of ATG MO replicated the phenotype of splice blocking MO (Figure E in S3 File). Of note, not only the co-injections with zebrafish *qdprb1* mRNA fully rescued the phenotype (Figure F in S3 File), but also with mouse *Qdpr* mRNA achieved morphological improvement. This result implies a conserved function of QDPR throughout the evolution of vertebrates.

### Metabolic alterations upon qdprb1 knockdown

**Fig 3:**
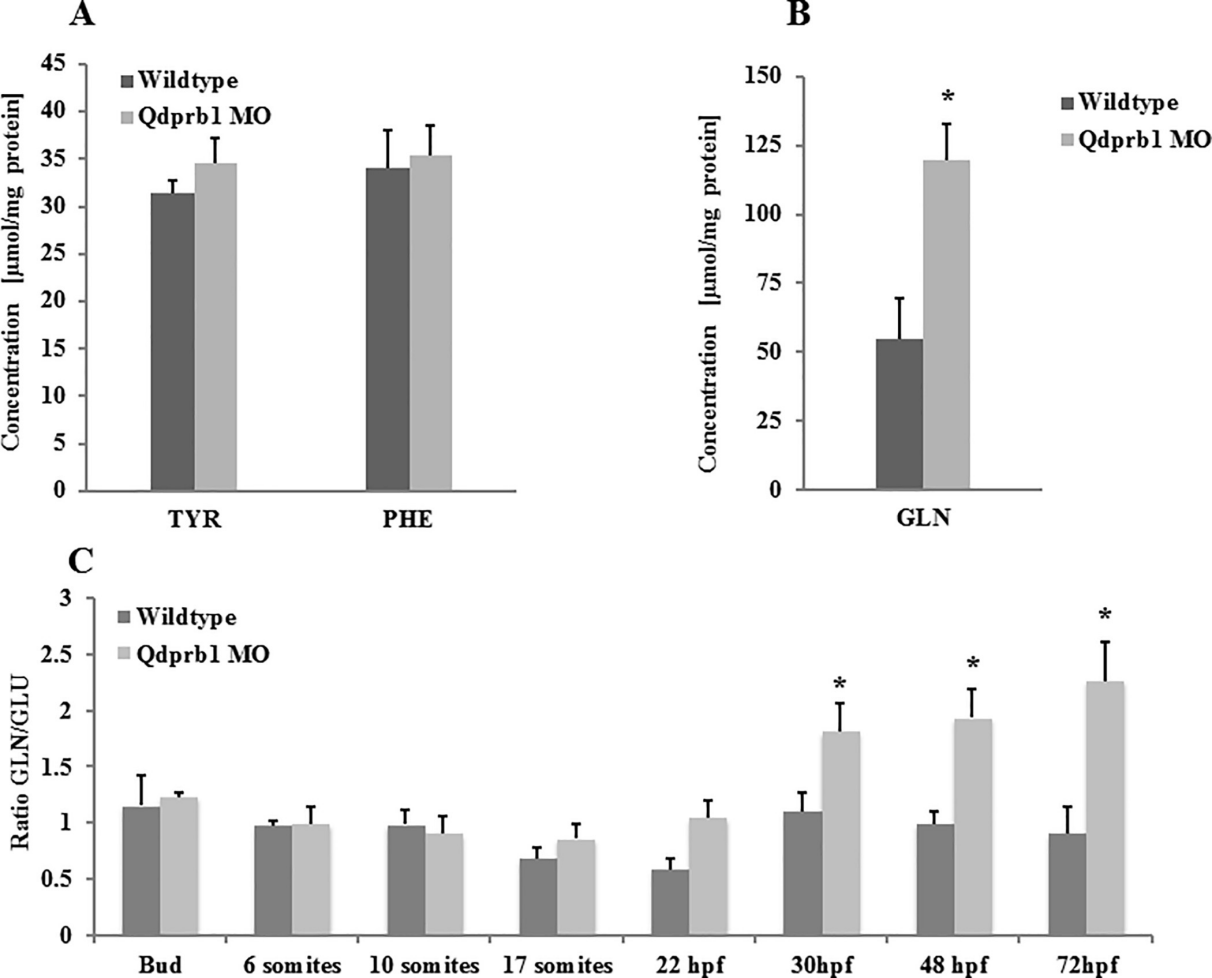
Biochemical analysis of Qdprb1 hypomorphic embryos. Amino acid analysis at 72 hpf shows reduced phenylalanine and unchanged tyrosine levels upon (A) as well as a strong increase of glutamine content (B) upon *qdprb1* knockdown. (C) Ratio of glutamine over glutamate in Qdprb1 hypomorphic embryos remains unchanged during gastrulation and early segmentation, but it starts increasing at the onset of the observed morphological phenotype (18 hpf) coinciding with the start of neuro- and gliogenesis. This pattern is not found in wildtype zebrafish.

Next, we assessed the biochemical consequences of qdprb1 reduction. Hypomorphic conditions did not affect phenylalanine levels at 72 hpf (Fig 3A). Intriguingly, the most prominent metabolic alteration we found in these embryos was a strong increase of glutamine (Fig 3B). Glutamine levels started rising at 18 hpf and steadily increased until 72 hpf (Fig 3C). Glutamine accumulation was not rescued by the co-injection with p53 MO and replicated upon ATG MO injection (Figure A, B in S4 File). This finding suggested that increased glutamine levels due to by *qdprb1* hypomorphic conditions may be the cause underlying the observed brain abnormalities in fish. Contrary to the “Trojan Horse” [33–35] implying toxicity by glutamate and ammonia in mitochondria, we did not observe any changes in glutamate or NH_3_ in *qdprb1* morphants (Figure C in S4 File) suggesting a different link between increased glutamine levels and brain size, or that these two alterations are caused independently from each other.

Due to the unexpected metabolic phenotype of *qdprb1* morphants, we next enzymatically characterized protein. We expressed and purified zebrafish Qdprb1 and human QDPR using the Champion pET SUMO Protein and Peptide Expression System (Thermo Fisher Scientific). In the subsequent enzyme assay both proteins reduced BH_2_ to BH_4_ confirming that zebrafish Qdprb1 has indeed *in vitro* properties of a dihydropteridine reductase (Qdprb1, Vmax 21 mU/mg, Km 50μM; QDPR, Vmax 15mU/mg, Km 30μM).

### Qdprb1 depletion increases proliferative markers

**Fig 4:**
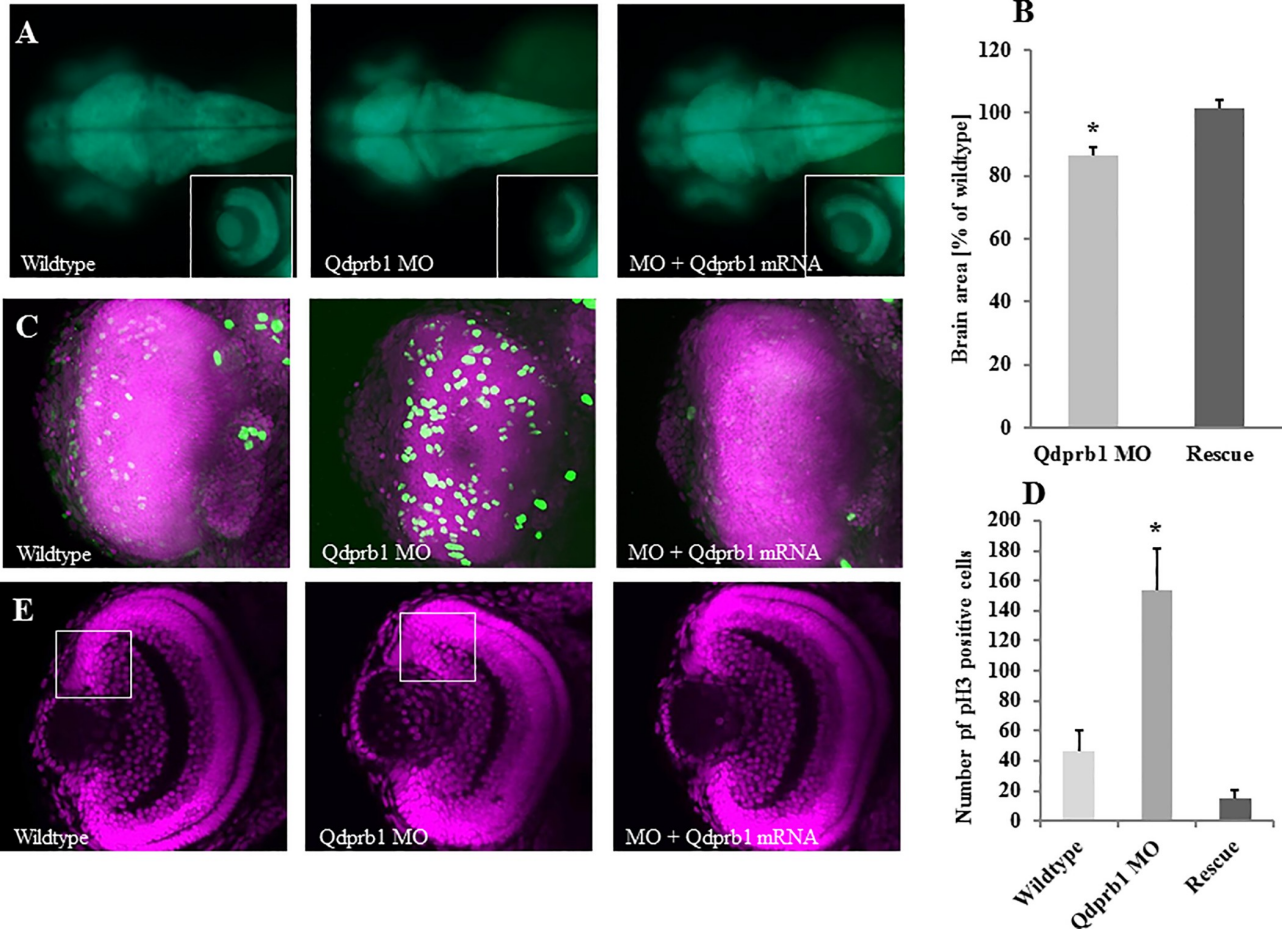
Reduced brain size but increased number of pH3-positive cells upon *qdprb1* knockdown. (A) Dorsal views, anterior to the left. At 3 dpf tg(HuC/D:GFP) transgenic zebrafish show a decreased size of the optic tectum and eye upon *qdprb1* knockdown, which can be rescued by co-injecting *qdprb1* mRNA. The insets show dorsal views of the left eye, which is reduced in size but still layered upon qdprb1 suppression. This phenotype is rescued upon co-injection of *qdprb1* mRNA. The overall GFP signal remains unchanged. (B) The brain area of Qdprb1 hypormorphic embryos is reduced by about 15% compared to wildtypes and rescued by co-injection of *qdprb1* mRNA. (C) Z-stack overlays of DAPI (pink) and pH3 (green) staining of dorsally imaged retinas reveal an increased number of pH3-positive retinal cells in Qdprb1 hypormorphic embryos (3 dpf), which are not restricted to the CMZ like found in wildtype retinas. Number and distribution of pH3-positive cells is restored by co-injection of *qdprb1* mRNA. (D) Statistical analysis shows a significant increase in pH3 positive cells upon *qdprb1* knockdown (n = 6) in comparison to wildtype zebrafish (n = 7), which could be rescued by addition of *qdprb1* mRNA (n = 3). (E) Z-confocal image of DAPI staining (pink) of the retina at 3 dpf shows retinal layers although decreased overall size and broadened CMZ in *qdprb1* hypomorphic embryos.

In line with its brain specific mRNA expression, *qdprb1* hypomorphic embryos exhibit a brain specific morphological phenotype. Indeed, for instance spinal cord and lateral line neurons marked in the transgenic line tg(NBT/lyn:GFP) appeared unaffected (Figure A in S5 File). Early marker genes for the midbrain and mid-hindbrain boundary such as *otx2* and *Wnt1* only showed a reduction in the size of their expression domains suggesting that early patterning of *qdprb1* expressing brain areas is unaffected (Figure B, C in S5 File). Consistently, *qdprb1* knockdown in tg(HuC/D:GFP) transgenic embryos expressing GFP in developing neurons confirmed that brain patterning and neuronal development occurred (Fig 4A and 4B). In contrast, sizes of the brain, most prominently of the optic tectum, and of the eye at 72 hpf were reduced, which could be rescued by co-injection of Qdprb1 mRNA (Fig 4A and 4B).

As early brain patterning appeared to be unaffected, we next investigated subsequent cellular processes to explain the observed microcephaly in *qdprb1* hypomorphic embryos. Since we already excluded p53 mediated apoptosis, we focused our attention on cell proliferation. Indeed, we found an increased number of cells positive for the mitotic marker phospho-histone H3 (pH3) (72hpf) in the eye (Fig 3C and 3D) and the optic tectum (Figure D in S5 File), i.e. *qdprb1* expressing brain areas. Noteworthy, pH3 positive retinal cells were not restricted to the CMZ but were found throughout the retina. This is unlike wildtype retinal cells, which start to differentiate once leaving the CMZ, indicating that *qdprb1* influences the transition from precursor cell proliferation and differentiation. DAPI staining of the retina of Qdprb1 hypomorphic embryos at 3 dpf showed that individual layers remained intact but the overall size was decreased and the CMZ was broadened (Fig 4E). The characteristic pH3 expression and localization in brain and eye as well as retina size and CMZ width was restored by *qdprb1* mRNA injection (Fig 4C, 4B and 4D). In conclusion, *qdprb1* knockdown does not influence major early brain patterning processes but instead causes a 4-fold increase of pH3 positive cells in the eye and optic tectum suggesting a function of Qdprb1 in promoting cell differentiation proliferation.

**Fig 5:**
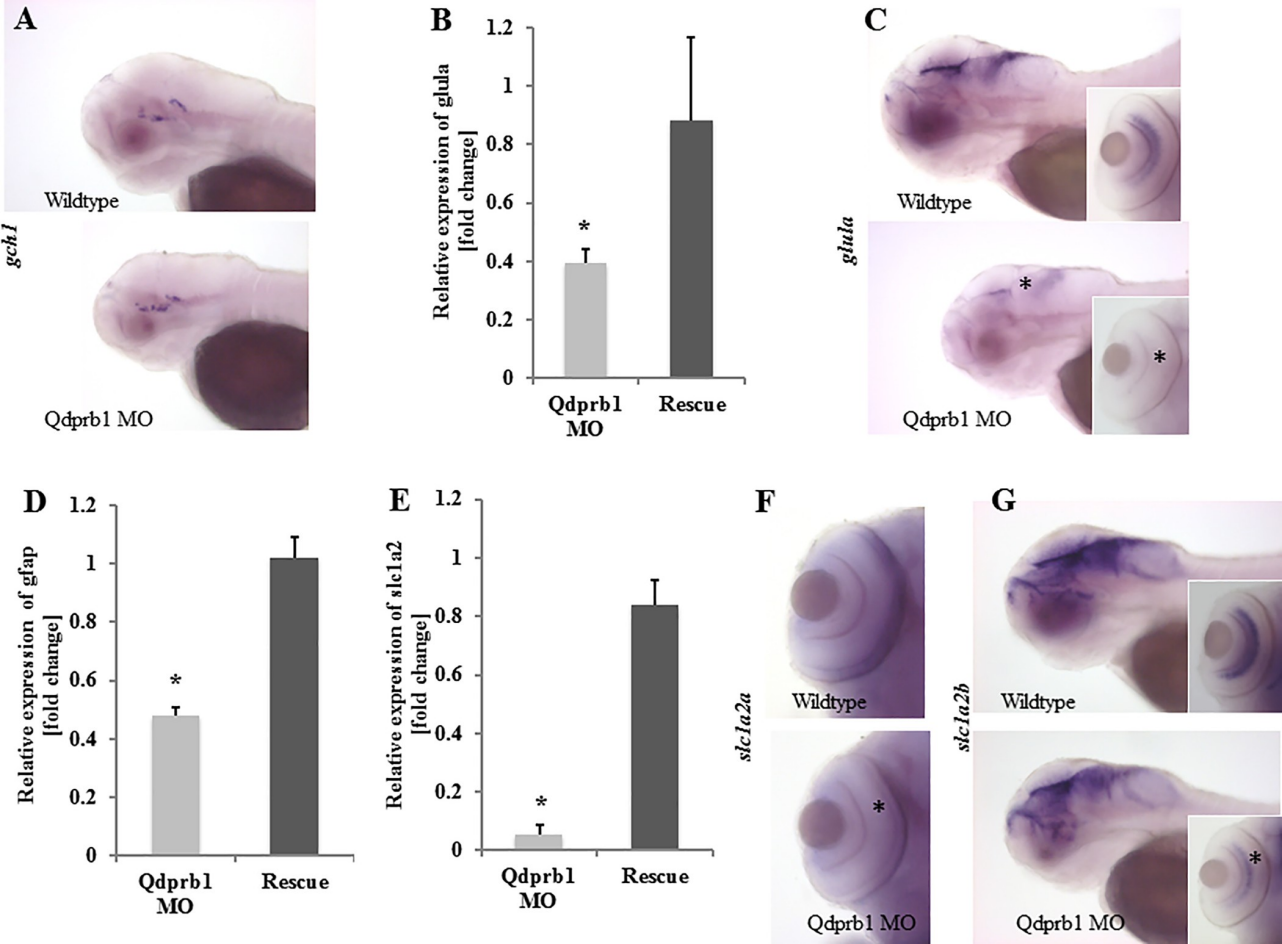
Expression of astroglial markers at 72hpf. (A) Lateral views with anterior to the left. WISH of the BH_4_ *de novo* synthesis pathway initiator and dopaminergic neuron marker, *gch1*, shows unchanged staining upon *qdprb1* knockdown. (B) RT-qPCR analysis reveals strongly reduced expression of g*lula* in *qdprb1* hypomorphic embryos, which is rescued by *qdprb1* mRNA co-injection. (C) Lateral views with anterior to the left. This finding is corroborated by WISH experiments highlighting that *glula* expression is lost in the eye (asterisk in inset) and reduced in the mid/hindbrain (asterisk). (D) Also g*fap* expression is reduced by *qdprb1* knockdown and can be rescued by *qdprb1* mRNA co-injection. (E) RT-qPCR analysis of astrocytic glutamate transporters show an almost complete loss of *slc1a2a*, which is confirmed by WISH (F)–dorsal views focused on the eye. Asterisk indicates the effect on *slc1a2a* expression. *Slc1a2a* expression is normalized in *qdprb1* mRNA co-injected embryos (E). (G) Lateral views with anterior to the left. WISH of s*lc1a2b* reveals mildly reduced staining in midbrain and eye (inset), confirming that both SLC1A2 homologues are affected in Qdprb1 hypomorphic embryos.

Due to the apparent microcephaly upon *qdprb1* knockdown, an increased number of pH3 positive cells was rather surprising. Nevertheless, defects of neural stem cell differentiation can also result in microcephaly [36]. By RT-qPCR expression analysis in 26 hpf *qdprb1* morphants we indeed found significant down regulation of genes critical for neurogenesis in brain and eye (*elavl3*, fold change 0.60 +/- 0.09; *neurod1*, fold change 0.63 +/- 0.02; *notch1a*, fold change 0.64 +/- 0.09; *rx2*, fold change 0.38 +/-0.04), whereas genes of cell proliferation were up-regulated (*myca*, fold change 1.80 +/- 0.32; *ccnd1*, fold change 1.40 +/- 0.19). Reduction of *gfap* expression (fold change 0.63 +/- 0.07) suggested that glial cell differentiation was also impaired. At 72 hpf neuronal markers suggested that dopaminergic neurons developed normally. This notion was corroborated by WISH staining of *gch1* giving a similar expression pattern as found in wild-type zebrafish (Fig 5A). Expression of the neuronal differentiation genes *neurod1* (fold change 0.47 +/- 0.12), the glial marker *gfap* (fold change 0.47 +/- 0.03), the eye development genes *rx1* (fold change 0.50 +/- 0.24) and *rx2* (fold change 0.71 +/- 0.05) were still decreased, whereas the pro-proliferative genes *ccnd1* (fold change 1.62 +/- 0.27) and *myca* (fold change 1.41 +/- 0.23*)* remained up-regulated.

### Qdprb1 knockdown depletes glial cell markers in the developing brain and eye

In mammals, astrocytes are the keepers of brain glutamine levels, removing glutamate from the synaptic cleft and shuttling it back to neurons in the form of glutamine. While teleosts lack typical star-shaped astrocytes, they contain radial glia that express specific astrocytic markers, such as g*lutamine synthetase a* (*glula*), and *gfap* [37, 38], suggesting that these cells have a similar function to astrocytes. Radial glial cells positive for astrocytic markers will further collectively be considered as astroglia in this manuscript.

To better understand the link between *qdprb1* knockdown and the rise of glutamine levels, we studied the expression of genes critical for cerebral glutamine/glutamate cycle in 72hpf *qdprb1* morphants, i.e. the stage with highest glutamine accumulation. Expression of the neuronal *glutaminase a* (*glsa*) was not affected (fold change 0.83 +/- 0.12) in *qdprb1* morphants, whereas its astrocytic counterpart, *glula*, was strongly down regulated (Fig 5B). This finding was confirmed in WISH experiments, showing decreased expression of *glula* in the inner retinal layer of the eye and the proliferative region of the midbrain (Fig 5C). Furthermore, the expression of second astrocytic marker Gfap depends on Qdprb1 function suggesting a functional role for qdprb1 in astroglia development (Fig 5D).

In mammals, SLC1A3 (EAAT1) and SLC1A2 (EAAT2) mediate the astrocytic glutamate uptake. The zebrafish orthologues of SLC1A2 display a distinct expression pattern with *slc1a2a* being present in the ONL and *slc1a2b* being expressed in the astroglia of the eye and brain [39]. RT-qPCR analysis and WISH staining showed an almost complete loss of *slc1a2a* (Fig 5E and 5F). Relative mRNA levels of *slc1a2b* were unchanged but WISH experiments showed a reduced expression in eye and midbrain (Fig 5G). In analogy to Slc1a2a/b, the two zebrafish orthologues of SLC1A3 show a distinct expression pattern with only Slc1a3b being expressed in brain tissue. Slc1a3b expression was unaltered in Qdprb1 hypomorphic embryos (Figure A, B in S6 File). Of note, the Genbank ID HM138691.1 used by [39] refers to *slc1a2a* mRNA, yet the annotated nucleotide sequence is identical to the one of the paralogue *slc1a2b* in the RefSeq database (used in our study). The same discrepancy occurs for *slc1a3a*.

Next, we analyzed expression of cerebral glutamine transporters. In mammals, system A (SLC38A1, 2) and system L (SLC7A5, 8) are responsible for the uptake of glutamine in neurons [40] but their role in the zebrafish brain is virtually unknown. We analyzed expression of *slc7a5* and s*lc38a2* in *qdprb1* morphants by RT-qPCR showing a mildly reduced expression of the latter transporter (Figure C in S6 File).

In summary, our data indicate a function for qdprb1 in the regulation of genes involved in brain glutamine metabolism and astroglia development.

### Effects of chronic exposure to high glutamine

**Fig 6:**
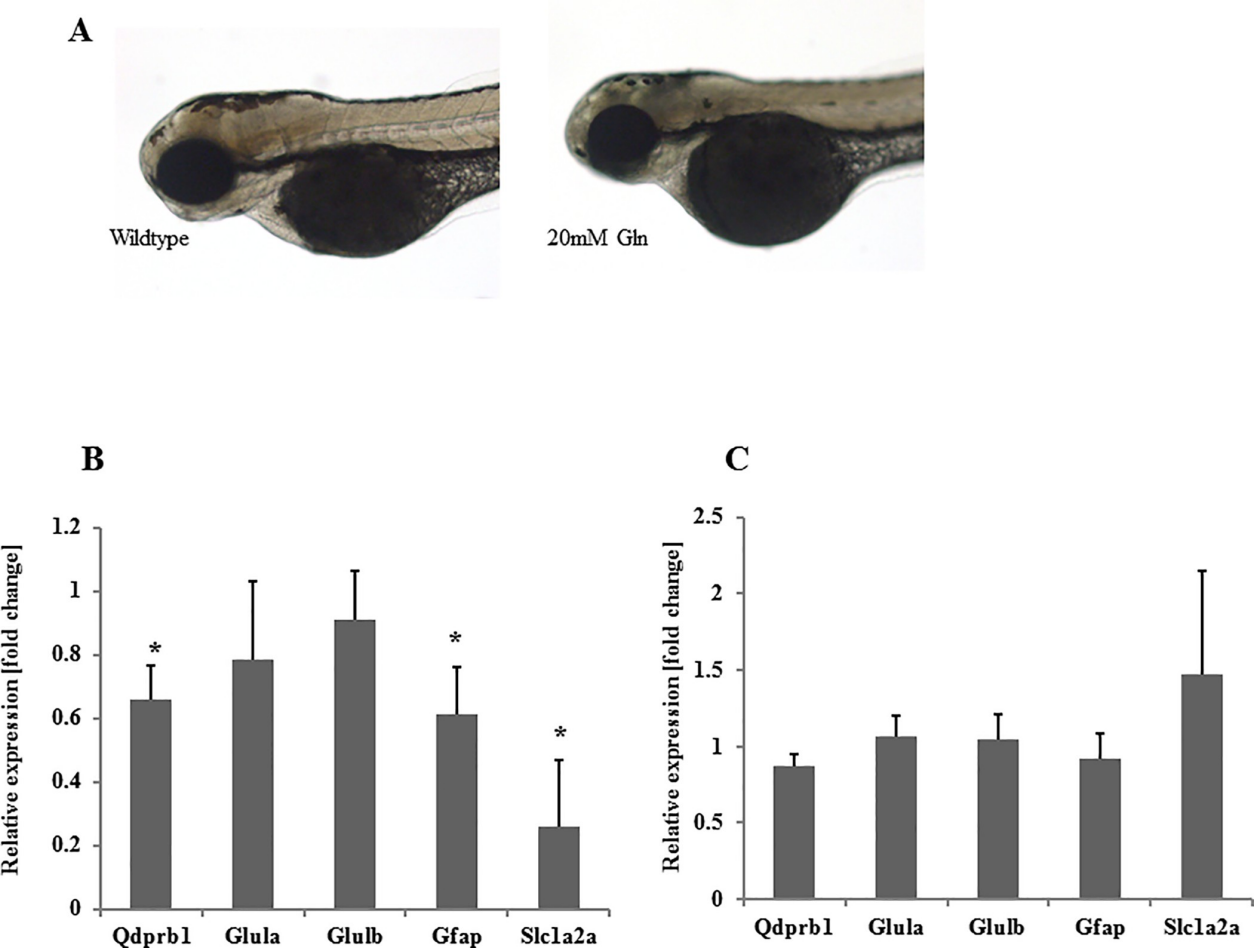
Glutamine exposure partially mimics *qdprb1* knockdown. (A) Lateral views with anterior to the left. Zebrafish exposed to 20 mM glutamine from sphere stage till 72 hpf display smaller heads and eyes. (B) RT-qPCR analysis zebrafish early exposed to glutamine (20 mM) shows reduced expression of *qdprb1*, *gfap*, and *slc1a2a*. (C) Late exposure to glutamine starting at 48 hpf does not affect the expression of these genes.

To shed more light on the mechanism of glutamine accumulation in Qdprb1 hypomorphic embryos, we assessed whether exposing zebrafish to high glutamine levels may induce a similar phenotype. Early developmental exposure (i.e. 5hpf to 72hpf) to high concentrations of glutamine (20 mM) resulted in developmental defects (Fig 6A), including small size of the head. RT-qPCR analysis revealed down regulation of *gfap* and *slc1a2a* expression as well as of *qdprb1*, though the latter effect was less pronounced (Fig 6B). Beside of highly increased cellular glutamine levels (wt, 55.93 +/- 1.62 μmol/mg; exposed, 469.7 +/- 283.53 μmol/mg) we also found ammonia accumulation (wildtype, 64,5 +/- 3,17 μmol/mg; exposed 255.7 +/-147.72 μmol/mg) in treated zebrafish, which is not found in *qdprb1* morphants. Later developmental exposure (i.e. 48hpf to 72 hpf) to 20 mM glutamine (Fig 6C) or exposure to lower glutamine levels (1 mM) did not induce a phenotype. Finally, we tested the glutamine synthase inhibitor L-methionine sulfoximine *qdprb1* morphants. It indeed reduced glutamine concentrations providing more evidence for a link between Qdprb1 activity and glutamine homeostasis, but failed to rescue the observed morphological phenotype.

Overall these experiments suggest a possible regulatory feedback between qdprb1 and glutamine metabolism in early phases of development as well as a role for glutamine in astroglia development.

### Glutamine in patients with DHPR deficiency

In case of a 1.5-year-old so far untreated female patient with DHPR deficiency, we documented an increase of glutamine in CSF (760 μmol/l (control range: 373–556 μmol/l)), while plasma levels remained normal. Clinically the patient presented a severe neurological picture with infantile Parkinsonism, epilepsy and brain atrophy. In follow-up analyses after treatment initiation with neurotransmitter precursors, folinic acid and phenylalanine reduced diet glutamine levels had normalized in CSF.

## Discussion

QDPR is required for the regeneration of BH_4_ that is a critical co-factor for the generation of dopamine and serotonin. Defects in the synthesis and recycling of BH_4_ lead to severe infantile Parkinsonism. In comparison to defects in BH_4_ biosynthesis, patients with inborn variants of *QDPR* present a higher frequency of severe neurological symptoms, including muscular hypotonia, dystonia, microcephaly, epilepsy and brain atrophy [11, 12]. The aim of our study was to establish a suitable model to investigate the pathophysiology of QDPR deficiency. To this end, we characterized expression and function of the three zebrafish QDPR homologues Qdpra, Qdprb1, and Qdprb2.

Amongst these three genes, only Qdpra and Qdprb1 appear to have a zygotic function during embryonic development, while Qdprb2 may have a maternal contribution as judged by its expression.

At early stages (up to 24hpf), *qdpra* is expressed in the eye and neural crest cells. Later on (120hpf) its expression is mainly defined to the liver. We find that Qdpra regulates melanin content and pigment size, overall linking this paralogue to melanin synthesis. The temporal expression pattern of *qdpra* suggests that the requirement of BH_4_ recycling for melanin synthesis is restricted to the early stages of melanophore development from neural crest cells, which has been shown for a number of genes [17]. Overall, *qdpra* expression correlates with *gch2*, the second paralogue of the *de novo* synthesis pathway initiator GCH, that is also expressed in pterin synthesizing xantophores [41] and BH_4_ dependent melanophores [42]. The lack of *qdpra* expression in dopaminergic or serotonergic neurons, as labeled by *gch1* and *th* respectively, makes it unlikely that BH_4_ recycling in these cells involves this *qdpr* paralogue. The observed hyperphenylalaninemia in *qdpra* hypomorphs is in line with the biochemical phenotype of patients and likely reflects the requirement of BH_4_ recycling for Pah activity. Accordingly, we find coexpression of *qdpra* and *pah* in the zebrafish liver.

Unlike the other two *qdpr* homologues, *qdprb1* is localized in the zebrafish brain, more specifically in the proliferative regions of the eye (CMZ) and optic tectum as well as in the mid-hindbrain boundary. This *qdprb1* expression in proliferative cells appears conserved and is also seen in proliferative brain regions of Medakafish [43] and embryonic mice [44]. Noteworthy, the proliferative regions of the eye and OT are genetically highly similar [45]. They harbor stem cells that differentiate into all glia and neurons required in the neuronal network [46, 47] and stay proliferatively active throughout development. Suppression of *qdprb1* results in microcephaly. While early brain patterning appears not affected, we observed an increased number of undifferentiated pH3-positive cells in the eye and OT. P53 mediated apoptosis was not enhanced. Further, we show up-regulation of pro-proliferative genes and down-regulation of genes associated with neural and glial differentiation. Our data suggest that qdprb1 influences the generation of neurons and astroglia from neuronal stem cells in the eye and OT. If qdprb1 function is suppressed, precursor cells fail to exit the cell cycle on time and remain undifferentiated. Indeed, the expression of proliferation promoting genes *myca* and *ccnd1* stayed up-regulated at 72 hpf. This suggests that the block of neural stem cell differentiation persisted during the observed time window of development. Of note *myca* has been reported to play also a role in the maintenance of the neural stem cell pool in the optic tectum [45]. Upon *qdprb1* knockdown the astroglia markers *gfap* and *glula* remained strongly reduced during the studied time window of development, while expression of some neuronal markers were unchanged indicating a more pronounced influence on gliogenesis. Also in the murine cerebellar cortex, Qdpr may play a role in glia cell development and/or function as it is strongly expressed glial cells [48].

Neuronal and glial differentiation starts with the switch of neural progenitor cells from symmetric to asymmetric cell division under the influence of pro-neuronal or pro-glial genes. Our data suggests that *qdprb1* knockdown interferes with the underlying signaling network. Although the underlying mechanism remains to be elucidated, the observed changes in gene expression can explain the microcephaly of *qdprb1* hypomorphs. Indeed, there is more evidence linking BH_4_ to cellular proliferation and differentiation. Proliferation of erythroid cells has been shown to be dependent on BH_4_ availability [49], while BH_2_ application blocked differentiation of these cells [50]. Using the pheochromocytoma cell line PC12 Anastasiadis et al. found that cellular BH_4_ levels influence c-Myc expression [51]. Further, tetrahydro-4-aminobiopterin, a structure analog of BH_4_ and NOS inhibitor, blocks dentritic cell-mediated T cell differentiation independent of NO activity suggesting an alternative and novel BH_4_-dependent signaling pathway [52]. Though these studies could not identify the underlying mechanism, they found no evidence that the observed effects are based on BH_4_ functioning as co-factor for PAH, TH, TPH or NOS. Of note, BH_2_ exposure of zebrafish tested in our study did not affect embryonic development (up to 1 mM in tank water) suggesting that the lack of qdprb1 but not of BH_2_ recycling induces the underlying pathomechanism. It needs to be addressed in future studies how and whether BH_4_ / BH_2_ directly or, as suggested by our study, via QDPR influence cell proliferation and differentiation.

The most prominent biochemical phenotype upon *qdprb1* knockdown was a strong increase of glutamine levels. Considering the brain-specific expression of *qdprb1*, this finding indicated impairment of cerebral glutamine homeostasis, which, at least in mammals, is regulated by astrocytes. In line with this notion several genes involved in the astrocytic glutamate uptake and glutamine synthesis were strongly affected in *qdprb1* hypomorphs. Microglia and oligodendrocytes have been identified and characterized in zebrafish [53, 54], whereas teleosts in general lack typical star-shaped astrocytes [53]. Studies indicate that astroglia of zebrafish mediate axon guidance in early embryo development [55], have regenerative capacity, and can replace damaged or lost neurons [56–58]. Expression of typical astrocytic markers (e.g. *glul*, *gfap* and *aqp4*) suggests that further functions of mammalian astrocytes including glutamate recycling are conserved [37, 53]. To get first insights into whether the reduction of glial markers was due to the observed increase of glutamine, we exposed wildtype zebrafish to different glutamine concentrations. This treatment resulted in reduced expression of *qdprb1*, *gfap*, and *slc1a2a* partially mimicking the *qdprb1* knockdown phenotype. However, unlike observed in qdprb1 hypomorphic conditions, treated zebrafish also displayed elevated ammonia levels. One may speculate that this finding supports the “Trojan horse” theory [33–35] underlying the observed phenotype, i.e. induced brain damage by glutamine being shuttled to mitochondria and broken down into glutamate and ammonia, which will result in free radical production and swelling of the mitochondria. It must be taken into account that the glutamine treatment affects the entire embryo rather than only a few cell populations in *qdprb1* knockdown embryos. Therefore, additional, more precise approaches are needed to explore the potential link between qdprb1, glutamine and glial development. If for instance astroglia cells in teleosts are found to be the sole source for cerebral glutamine production, then the effects of *qdprb1* knockdown (glutamine increase and glia development) are two separate events. Alternatively, glutamine generation may derive from elevated pterin deaminase activity due to disturbed BH_4_ recycling. This protein has mainly been studied in prokaryotes but it was also found in rat liver and zebrafish, and is thought to regulate BH_4_ metabolism [59].

Cohort studies on BH4 deficiencies do not report increased glutamine levels in CSF or blood [11]. Retrospective evaluation of CSF samples of BH_4_ deficient patients in our centre documented high glutamine levels in one young, untreated, and severely affected female DHPR-deficient patient with infantile Parkinsonism, epilepsy and brain atrophy. The fact that glutamine levels normalized after onset of treatment may suggest that glutamine levels correlate with disease severity and are normal in treated or less severely affected patients. Thus, there may well be a link between DHPR-deficiency and glutamine levels, which is reproducible in our fish model but had so far been overlooked in patients.

## Conclusion

This study is the first to characterize the expression and function of the three zebrafish QDPR homologues *qdpra*, *qdprb1*, and *qdprb2*. While we could not identify a zygotic function for *qdprb2* in embryonic development, *qdpra* executes the expected BH_4_ recycling function in melanin producing cells and in the liver. Our results further suggest that *qdprb1* is required for neuronal and glial differentiation of neural progenitor cells emerging from the CMZ and OT.

## Supporting information

S1 File Developmental expression profiles of *qdprb2*, *qdpra*, and *qdprb1*. Relative mRNA expression levels during different developmental stages, in reference to 50% epiboly, of Qdprb2 (A), Qdpra (B), Qdprb1 (C).
(TIF)

S2 File Characterization of Qdpra. **(**A) Lateral views, anterior to the left. Expression of *Pah* at 72 hpf is found in retinal pigment epithelium (red arrow), fin bud (blue arrow) and liver (white arrow). (B) RT-PCR shows loss of exon 3 upon splice blocking MO injection. (C) Lateral views, anterior to the left of 72 hpf embryos. Aberrant pigmentation of Qdpra hypomorphic embryos can be rescued by co-injection of *qdpra* mRNA.
(TIF)

S3 File Characterization of Qdprb1. **(**A) Lateral views, anterior to the left of 72 hpf embryos. *p53* knockdown does not rescue the microcephaly phenotype of Qdprb1 hypomorphic embryos. (B) RT-PCR confirms the predicted inclusion of intron 3 upon injection of the splice blocking MO resulting in a strong reduction of correctly spliced mRNA (RT-qPCR, C). (D). Qdprb1 morphant phenotypes using low concentrations of each MO and a combination of both showing a synergy effect between both. (E) Lateral view, 72hpf ATG MO Qdprb1 injected embryos reproduce the phenotype of Splice MO Qdprb1 hypomorphic embryos. (F) Lateral views, anterior to the left. Co-injection of *qdprb1* mRNA in Qdprb1 hypomorphic embryos rescues brain development.
(TIF)

S4 File Biochemical phenotype upon Qdprb1 knockdown. (A) *P53* knock down does not prevent glutamine accumulation in Qdprb1 hypomorphic embryos. **(**B) MO-mediated blocking of Qdprb1 translation also results in glutamine accumulation. (C) Glutamine accumulation in Qdprb1 hypomorphic embryos is not linked to increased glutamate or ammonia generation.
(TIF)

S5 File Brain development in Qdprb1 hypomorphic embryos. (A) Lateral views, anterior to the left of 72 hpf embryos. *Qdprb1* knock down does not affect development of for instance motor neurons (left) and lateral line organ (right) in tg(NBT/lyn:GFP) transgenic zebrafish (red arrows). (B) Lateral views, anterior to the left and (C) dorsal views with anterior to the left at 26 hpf stained for *wnt1* (B) and *otx2* (C) expression show unchanged expression patterns but reduced size of the positively stained region upon *qdprb1* knockdown. (D) Z-stacks of DAPI (pink) and pH3 (green) staining of the optic tectum reveals an increase of proliferating cells in 72 hpf Qdprb1 hypomorphic embryos.
(TIF)

S6 File WISH and RT-qPCR of glutamate and other solute carrier transporters. RT-qPCR (A) analysis and WISH (B; lateral views, anterior to the left) shows unchanged expression of *slc1a3b* in Qdprb1 hypomorphic embryos. (C) Further, expression of *slc7a5* remained unchanged and of *slc38a2* was reduced in these zebrafish.
(TIF)

S1 Table List of studied genes and used primer pairs. (TIF)
