# Supplementary figures and images for: QDPR homologues in Danio rerio regulate melanin synthesis, early gliogenesis, and glutamine homeostasis
Source: PLoS One. 2019 Apr 17;14(4):e0215162. doi: 10.1371/journal.pone.0215162 (PMC6469847; doi:10.1371/journal.pone.0215162)

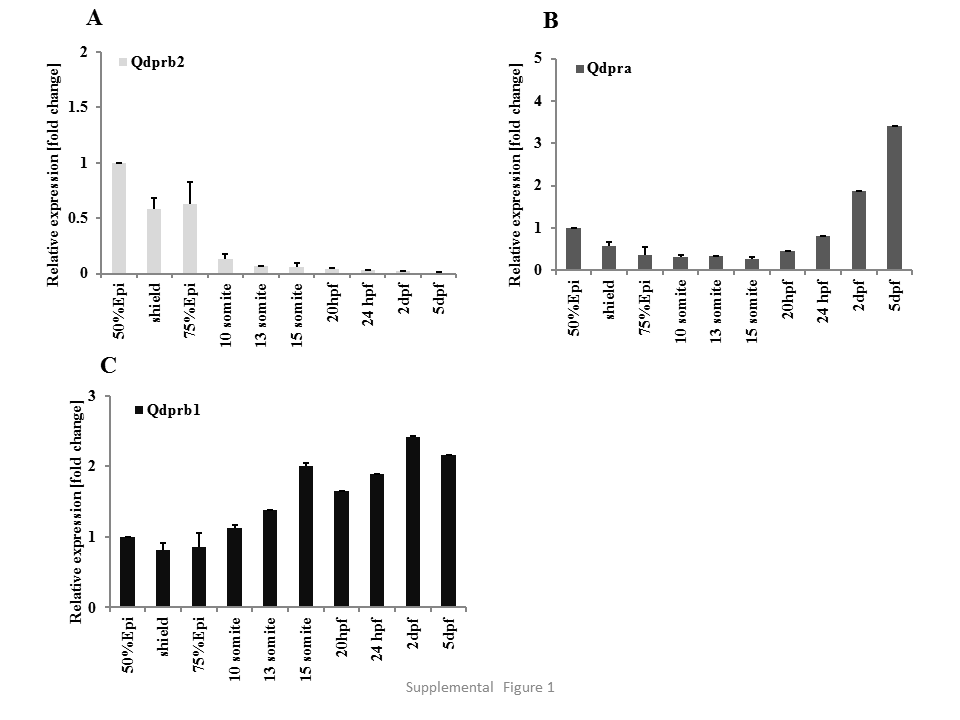

Supplement: S1 File — Relative mRNA expression levels during different developmental stages, in reference to 50% epiboly, of Qdprb2 (A), Qdpra (B), Qdprb1 (C). (TIF) [file pone.0215162.s001.TIF]

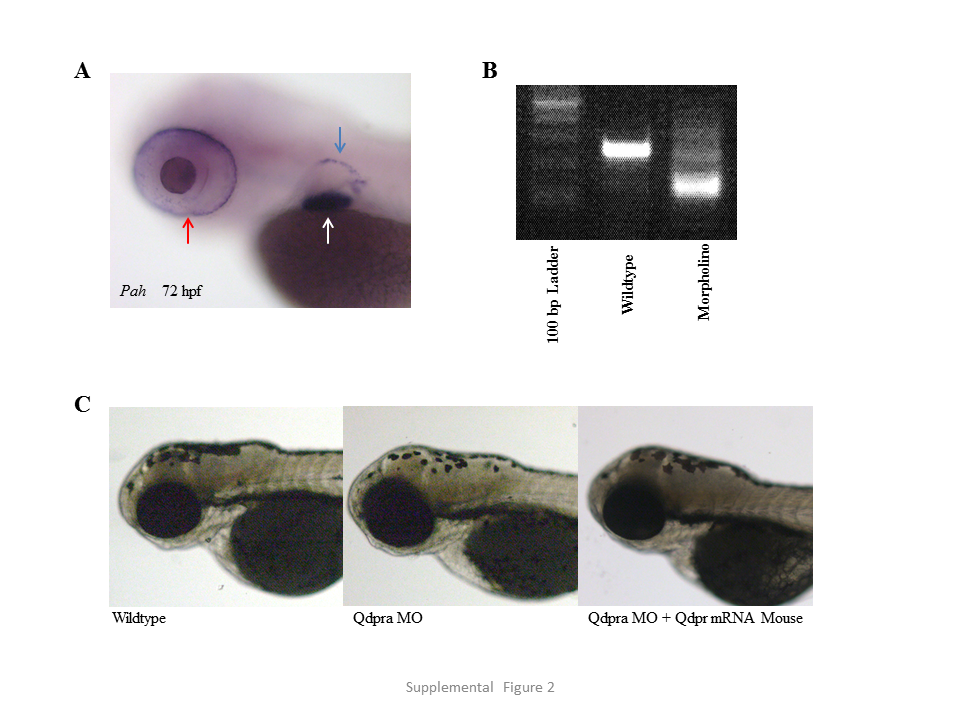

Supplement: S2 File — (A) Lateral views, anterior to the left. Expression of Pah at 72 hpf is found in retinal pigment epithelium (red arrow), fin bud (blue arrow) and liver (white arrow). (B) RT-PCR shows loss of exon 3 upon splice blocking MO injection. (C) Lateral views, anterior to the left of 72 hpf embryos. Aberrant pigmentation of Qdpra hypomorphic embryos can be rescued by co-injection of qdpra mRNA. (TIF) [file pone.0215162.s002.TIF]

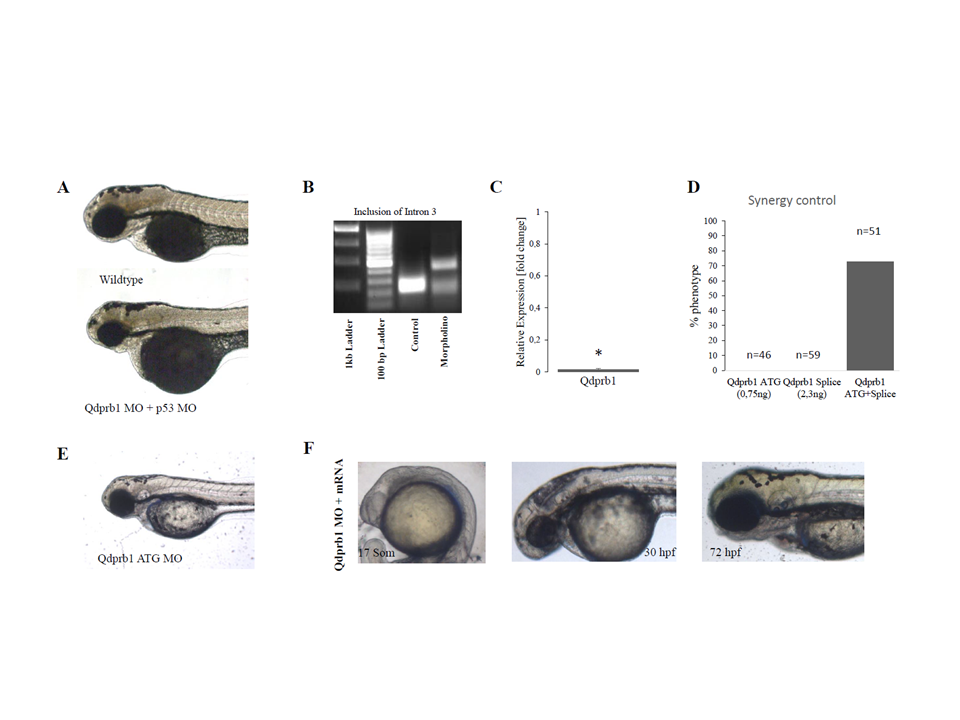

Supplement: S3 File — (A) Lateral views, anterior to the left of 72 hpf embryos. p53 knockdown does not rescue the microcephaly phenotype of Qdprb1 hypomorphic embryos. (B) RT-PCR confirms the predicted inclusion of intron 3 upon injection of the splice blocking MO resulting in a strong reduction of correctly spliced mRNA (RT-qPCR, C). (D). Qdprb1 morphant phenotypes using low concentrations of each MO and a combination of both showing a synergy effect between both. (E) Lateral view, 72hpf ATG MO Qdprb1 injected embryos reproduce the phenotype of Splice MO Qdprb1 hypomorphic embryos. (F) Lateral views, anterior to the left. Co-injection of qdprb1 mRNA in Qdprb1 hypomorphic embryos rescues brain development. (TIF) [file pone.0215162.s003.tif]

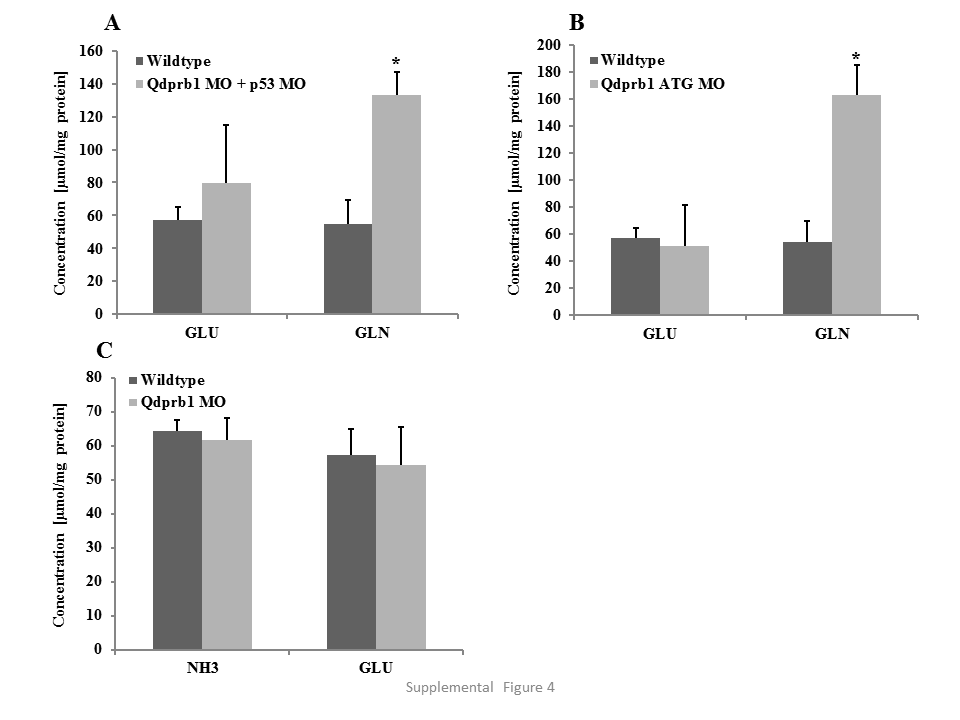

Supplement: S4 File — (A) P53 knock down does not prevent glutamine accumulation in Qdprb1 hypomorphic embryos. (B) MO-mediated blocking of Qdprb1 translation also results in glutamine accumulation. (C) Glutamine accumulation in Qdprb1 hypomorphic embryos is not linked to increased glutamate or ammonia generation. (TIF) [file pone.0215162.s004.TIF]

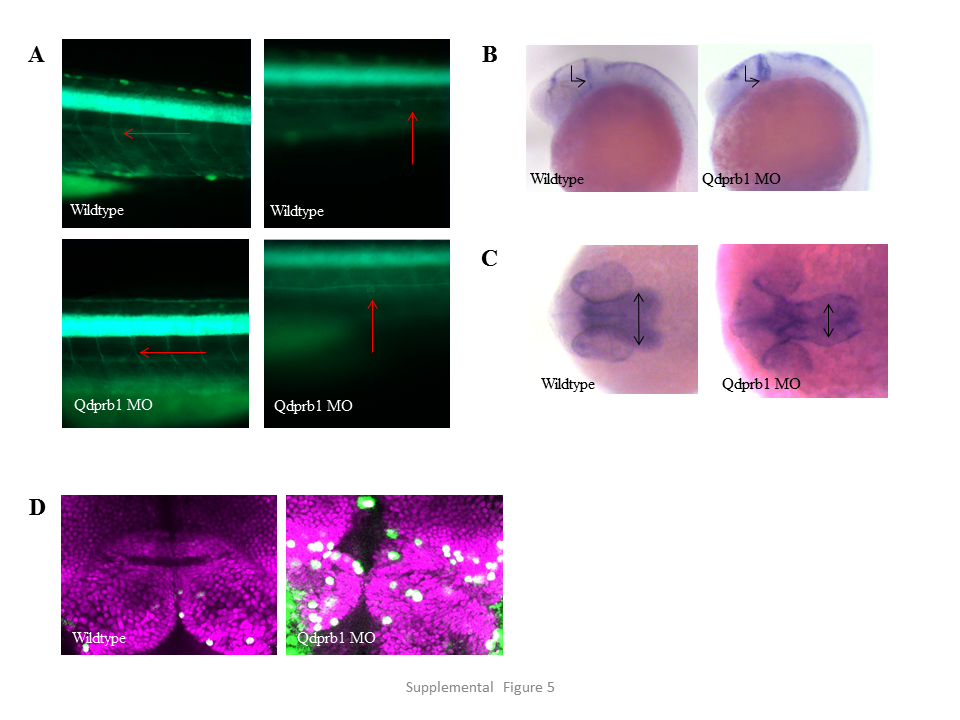

Supplement: S5 File — (A) Lateral views, anterior to the left of 72 hpf embryos. Qdprb1 knock down does not affect development of for instance motor neurons (left) and lateral line organ (right) in tg(NBT/lyn:GFP) transgenic zebrafish (red arrows). (B) Lateral views, anterior to the left and (C) dorsal views with anterior to the left at 26 hpf stained for wnt1 (B) and otx2 (C) expression show unchanged expression patterns but reduced size of the positively stained region upon qdprb1 knockdown. (D) Z-stacks of DAPI (pink) and pH3 (green) staining of the optic tectum reveals an increase of proliferating cells in 72 hpf Qdprb1 hypomorphic embryos. (TIF) [file pone.0215162.s005.TIF]

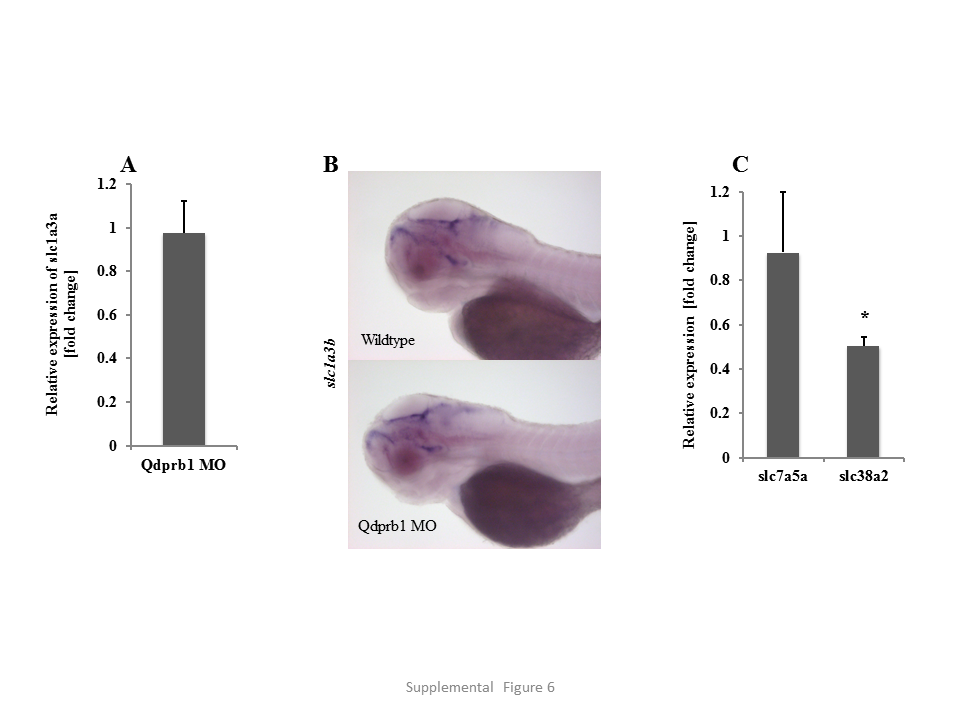

Supplement: S6 File — RT-qPCR (A) analysis and WISH (B; lateral views, anterior to the left) shows unchanged expression of slc1a3b in Qdprb1 hypomorphic embryos. (C) Further, expression of slc7a5 remained unchanged and of slc38a2 was reduced in these zebrafish. (TIF) [file pone.0215162.s006.TIF]

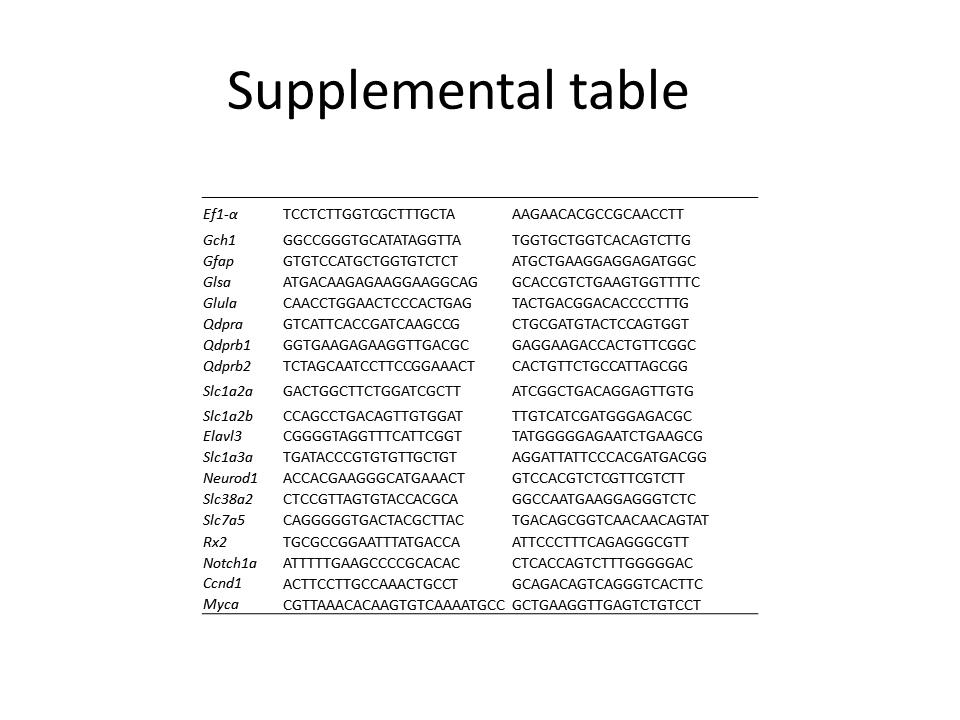

Supplement: S1 Table — (TIF) [file pone.0215162.s007.TIF]
